# Evaluating the awareness and knowledge of dyslexia among primary school teachers in Tshwane District, South Africa

**DOI:** 10.4102/ajod.v11i0.807

**Published:** 2022-04-28

**Authors:** Mary M. Makgato, Monicca Leseyane-Kgari, Madoda Cekiso, Itani P. Mandende, Rose Masha

**Affiliations:** 1 Department of Applied Languages, Faculty of Humanities, Tshwane University of Technology, Pretoria, South Africa

**Keywords:** dyslexia, reading, dyslexic learner, reading instruction, teacher knowledge

## Abstract

**Background:**

Many developed countries have made rapid strides in addressing issues related to dyslexia but in the developing countries like South Africa, it has not received adequate attention.

**Objectives:**

The study therefore sought to evaluate awareness and knowledge of dyslexia among primary school teachers working in the government sector.

**Methods:**

A phenomenological design was used and the study followed a mixed methods approach. The sample included 30 purposively selected primary school teachers. A questionnaire that consisted of true and false questions, closed-ended questions and open-ended questions was used to collect data. SPSS Version 22 and Excel Data Analyser 4 were used to analyse the quantitative data whereas the qualitative data was analysed thematically.

**Results:**

The results indicated that the primary school teachers had a basic awareness and knowledge of dyslexia. Many of them were found to be using limited strategies in order to teach learners with dyslexia in their classrooms.

**Conclusion:**

Based on the findings, recommendations such as early diagnoses through testing, parental involvement, conducive learning environment and teachers’ professional development regarding dyslexia were made.

## Introduction

Dyslexia is a common term which refers to specific reading difficulties which can be classified as a Specific Learning Disorder (SLD) (According to the American Psychiatric Association 2013). American Psychiatric Association considers SLD to be a type of neurodevelopmental disorder that impedes the ability to learn or use specific academic skills such as reading, writing or arithmetic. However, an SLD in reading is still commonly referred to as dyslexia in the literature. As such, the authors in this article make reference to dyslexia instead of SLD. Dyslexia is a specific neurobiological disorder characterised by difficulties in reading fluency, decoding and spelling skills resulting from a deficit in the phonological component of a language despite having received adequate instruction and having average intellectual level (Lonigan et al. 2013). In addition, Fawcett, Nicolson and Dean (1996) point out that:

[*C*]hildren with dyslexia often have associated deficits in certain related domains such as oral language acquisition, writing abilities, mathematical abilities, motor coordination, postural stability and dexterity, temporal orientation, visuospatial abilities and dexterity, and attention abilities. (p. 273)

Previous studies indicated that dyslexia is the most common learning disorder in children, but it has not received adequate attention in developing countries (Shetty & Sanjeev Rai 2014). This view is worrying in a South African context where the White Paper 6 (Department of Education 2001) seeks to establish ‘an education and training system that ensures that all learners with and without disabilities, pursue their learning potential to the fullest’.

Because South Africa has introduced the policy of inclusive education, our main concern is how far the government has gone in developing public school teachers to cope with a diversity of learning and teaching to accommodate learners with learning disabilities such as dyslexia. According to Artiles, Dorn and Christensen (2006:65), inclusive education is understood to refer to the placement of learners with special educational needs in mainstream settings, along with other learners without learning challenges. However, in the South African context, inclusive education refers to the capacity of ordinary local schools and Early Childhood Developmental Centres to respond to the needs of all learners (Booth 2015:2). In addition, McLeskey et al. (2004) are of the view that inclusive education establishes acceptable educational practices in general education schools by providing a range of educational services to assist all learners with special needs to learn to their fullest potential. In order to achieve this goal, teacher training for primary school teachers becomes paramount. Swanson and Hsieh (2009:1362) are of the view that primary school teachers play ‘an important role in the early identification of learners with dyslexia, and their awareness on dyslexia would be of assistance in the management of learners with dyslexia’. Therefore, teachers’ awareness and knowledge of dyslexia is contextually significant because any challenge with reading is likely to negatively affect the learner’s academic success.

There is considerable research supporting the claim that South Africa has a reading crisis (Howie et al. 2011; Rule & Land 2017; Spaull 2016; Willenberg 2018). For example, the poor performance by South African Grade 6 learners in the Southern and Eastern Africa Consortium for Monitoring Education Quality (SACMEQ) (2007) is a cause for serious concern. Southern and Eastern Africa Consortium for Monitoring Education Quality is a cross-national initiative consisting of 14 countries in southern and eastern Africa and it tests the numeracy and literacy skills of Grade 6 learners in each of the participating countries. The results of the SACMEQ study (2007) show that of the 15 countries that participated, South Africa came 10th in reading. This shows that South Africa was lagging behind much poorer African countries such as Tanzania and Zimbabwe (Rule & Land 2017; Van der Berg 2007). However, the focus of this study is on dyslexia as it pertains to reading and not mathematics.

The recent results of the 2016 Progress in International Reading Literacy Study (PIRLS) do not show any improvement as far as the reading ability of Grade 4 South African learners is concerned. The results indicate that 8 out of 10 South African Grade 4 learners cannot read. For example, South African Grade 4 learners could not locate and retrieve explicitly stated information or make straightforward inferences about events and reasons for actions (Spaull 2016; Willenberg 2018).

Although the authors of this study do not have any scholarly evidence linking the reading crisis in South Africa to dyslexia as a challenge, most international bodies such as the International Dyslexia Association and the British Dyslexia Association claim that about 10% to 15% of the population is affected by dyslexia. This claim is supported by Knight (2018) who declares that an estimated 5% to 10% of the worldwide population is said to have dyslexia and, accordingly, it is important that teachers understand what dyslexia is and how it affects their learners. These international bodies further claim that many more learners with dyslexia are not getting the help they need and worst of it all are not assessed by schools. The situation is made worse by the claim made by Khaliq, Ramsan and Aslam (2017:2) that ‘too often, learners with dyslexia remain undiagnosed throughout their school careers, labelled instead as lazy or disruptive learners’. Khaliq et al. (2017:3) further state that such ‘learners face the misery of failure, depression and an increased risk of suicide, delinquency and reoffending’. Similarly, Delany (2017:97) states, ‘many students in the mainstream schools do not receive optimum academic support and, as a result, struggle to keep up with their non-dyslexic peers’. In the South African context, the authors have observed that many learners who have reading challenges have not been assessed for dyslexia. Abd Rauf et al. (2018) as well as Silva Do Nascimento, Carneiro Rosal and Manchester De Queiroge (2018) allude to the fact that learners with dyslexia need specialised teachers.

The important question to ask is whether such specialised teachers exist in South Africa. Hence the authors decided to investigate primary school teachers’ awareness and knowledge of dyslexia. This is an important question to as against the background that if not diagnosed and relevant interventions provided accordingly at the early levels of schooling, dyslexia is likely to pose reading challenges to learners (Nalavany, Carawan & Brown 2011).

As already indicated above, despite dyslexia being recognised as a major educational challenge, it has not received sufficient support in developing countries like South Africa (Shetty & Sanjeev Rai 2014). Few studies that have been conducted in this regard reveal that teachers held a basic understanding of dyslexia and lacked the knowledge of the biological and cognitive aspects of dyslexia (Knight 2018; Shari & Narasimha 2015). Subsequently, Knight (2018) argues that:

[*E*]vidence-based teacher training, which informs teachers of the up-to-date research on the biological, cognitive, and behavioural aspects of dyslexia, is essential to combat misconceptions and ensure that teachers have more nuanced and informed understandings of dyslexia. (p. 207)

Khaliq et al. (2017) conducted a study in Pakistan and the findings revealed that teachers of the elementary schools from Lahore were not aware of the term dyslexia and only few of them were able to identify and manage it in their classrooms. This is a serious situation because teachers are supposed to be trained to screen for dyslexia so that appropriate referrals can be made. Teachers also play a significant role in early identification of such disorders in children.

Although many studies have been conducted in the developed countries such as United Kingdom (UK) and United States of America (USA), which have policies on dyslexia, few studies have been conducted in South Africa on the learners with dyslexia. The most similar study conducted in a South African context was conducted by Thompson (2013). The aim of Thompson’s study was to ‘assess teachers’ awareness levels of dyslexia, their perceptions of their ability to identify and manage dyslexia, and their perceptions of the adequacy of their pre-service and in-service training in dyslexia’. The results of the study indicated that teachers had adequate knowledge of dyslexia, and they believed they were able to identify and manage dyslexia and that they received little or no pre-service and in-service training in dyslexia. The authors of this article found contradictions in the findings of Thompson’s study. For example, if teachers believed that they received little or no pre-service and in-service training in dyslexia, then, on what academic grounds do they claim to have enough knowledge of dyslexia and able to identify and manage learners with dyslexia? Thompson’s study does not provide answers in this regard. In the current study, there is a little difference between awareness and knowledge. On the one hand, awareness refers to perceiving, feeling or being conscious of events, thoughts, emotions or sensory patterns while on the other hand, knowledge refers to facts, information and skills acquired through experience or education. We believe that awareness leads to knowledge or that there is no knowledge without awareness. Subsequently, the current study sought to provide answers to the following questions:

**Research Question 1**: What is the public primary school teachers’ level of awareness of the concept ‘dyslexia’?

**Research Question 2:** What is the teachers’ understanding of limitations brought about by dyslexia in teaching and learning?

**Research Question 3**: How do teachers navigate teaching and learning within reading difficulties brought about by dyslexia?

**Research Question 4:** To what extent are the teachers interested in dyslexia training?

### What is dyslexia?

According to Mattke (2021), dyslexia is a learning disorder that involves difficulty in reading due to problems in identifying speech sounds and learning how they relate to letters and words (decoding). The International Dyslexia Association and National Institutes of Child Health and Human Development offers a current definition of dyslexia as a specific learning disability that is neurological in origin. The International Dyslexia Association and National Institutes of Child Health and Human Development argue that dyslexia is not a disorder but a SLD. Many authors come to a consensus that dyslexia is linked to genes, which is why the condition often runs in families (International Dyslexia Association and National Institutes of Child Health and Human Development 2017; Shroff 2021; The International Dyslexia Association n.d.). Shroff (2017) is of the view that one is likely to have dyslexia if one’s other family members have it. It appears to be genetic and affects how the brain processes reading and language. The Clinic Guide to Raising a Healthy Child (2017) declares that the symptoms of some early clues of dyslexia may include late talking, learning new words slowly, challenges in forming sounds correctly, such as, reversing sounds alike, challenges in remembering or naming letters, numbers and colours, learning nursery rhymes as well as playing rhyme games. According to the International Dyslexia Association (n.d.), diagnostic evaluations of dyslexia often cover background information, including family history and early development, intelligence, oral language skills, word recognition, fluency skills, reading comprehension, vocabulary knowledge, decoding and phonological processing. Shroff (2021) states that the brain of learners with dyslexia has a hard time connecting letters to the sounds they make, and then blending those sounds into words. According to Shroff, to someone with dyslexia, the word ‘cat’ might read as ‘tac’ and because of these mix-ups, reading can be a slow and difficult process.

### The importance of teacher awareness and knowledge of dyslexia

In many countries, the role and functioning of schools are changing and so is what is expected of teachers (Davis & Watson 2000). In this regard, South Africa is no exception, especially with the introduction of inclusive education policy. In 2001, South Africa developed inclusive education in line with international trends and the social rights discourse. As a result, the policy document, Education White Paper No: 6 (2001) was established. This outlined and embraced the government’s obligation to provide a supportive inclusive education environment for learners with special needs (Sukhraj 2006). In the context of this study, inclusion is broadly defined as the process through which learners who might have previously been taught in a separate special education system due to learning challenges are now taught in an ordinary classroom. Despite the adoption of an inclusive education policy in South Africa, Peters (2007) believes that learners with dyslexia continue to be vulnerable. Donohue and Bornman (2014) concluded that the implementation challenges of inclusive education are attributable to two main factors, namely, the apparent lack of clarity in the policy and various issues around the poor implementation of the policy. At the heart of the poor implementation of inclusive education is the lack of teachers’ skills and knowledge in differentiating the curriculum to address a wide range of learning needed (Donohue & Bornman 2014). However, there are contradictions in the literature concerning the implementation efforts of inclusive education in South Africa. For example, Adewumi, Mosito and Agosto (2019) conducted a study on the experiences of teachers in implementing inclusion of learners with special education needs in Fort Beaufort District (South Africa). Their findings revealed that teachers accommodated learners with special education needs like dyslexia, despite the fact that some of them did not have the needed qualifications or training on learners with special education needs.

Despite the contradictions in the findings, consensus is that there is a need to develop teachers in ordinary schools so that they can cope with the learners with dyslexia in their classrooms. Supporting the idea of teacher development in South Africa, Lessing and De Witt (2007) point out that since 1994, a year in which South Africa transitioned from the system of apartheid to one of democracy, major changes have occurred in education policy. They further state that teachers have been challenged to attend to learners with barriers to learning in an inclusive classroom. According to Coetzer (2001), inclusive education, will only be effective if teachers are adequately prepared and equipped by means of professional development. Anderson, Case and Lam (2001) believe that during the moments of change in an education system, it is necessary to help teachers update their knowledge and skills to deal with change, on the one hand, and manage human resources better, on the other hand. However, findings of studies conducted by Prinsloo (2001) and Peters (2007) revealed that despite the introduction of an inclusive education policy in South Africa, learners with disabilities such as dyslexia remain vulnerable.

It has already been mentioned hitherto that many studies on learners with dyslexia have been conducted in developed countries like the UK and the USA. Subsequently, such countries have policies on dyslexia. Few studies have been conducted in the developing countries like South Africa on learners with dyslexia. Therefore, the authors separate reporting literature from developed and developing countries. Literature on dyslexia in the developed countries reveals that such countries are at an advanced stage about addressing dyslexia. For example, the Department for Education and Skills (2004) in the UK developed a framework for understanding dyslexia. This framework addressed the definition of dyslexia, theories of dyslexia, approaches, and programmes used by specialists.

In addition to the foregoing, several studies have examined teacher knowledge and awareness of dyslexia and generally found weaknesses in some areas of awareness and knowledge and strength in others (Elias 2014; Furnham 2013; Knight 2018). These studies come to a consensus that teachers’ awareness and knowledge of dyslexia is significant in developed countries for them to be able to help learners with dyslexia most effectively by implementing the best methods to help these learners. According to Dyslexic Action (2012:7), teachers who lack understanding of the nature of dyslexia run the risk of being unhelpful and use damaging comments that have long-lasting detrimental effects to the learners with dyslexia. It is thus important that teachers and schools have adequate understanding of dyslexia, as this understanding is likely to affect teachers’ practice. Knight (2018) is of the view that teachers’ awareness and knowledge about dyslexia is significant so that teachers could identify those learners at risk and can develop relevant interventions.

The results of a study conducted by Furnham (2013) on teachers’ understanding of dyslexia revealed that teachers were unsure about the neurobiological aspects of dyslexia. In a study conducted by Knight (2018), it was identified that there were 12 teachers who had been trained in special education, while only five teachers out of 143 indicated having experience in teaching learners with dyslexia. Knight (2018:4) observed that ‘teachers had basic awareness on dyslexia yet lacked the awareness on specific symptoms of dyslexia which are crucial in early identification of learners with dyslexia’. Moreover, the teachers were found lacking the ability to make adaptation in teaching materials and assessment to suit the needs of learners with dyslexia. Furnham (2013) conducted a study in the United Kingdom and the findings revealed that although the participants provided a relevant definition of dyslexia, they were not sure about the neurobiological aspects of dyslexia. Also commenting on the importance of teachers’ awareness and knowledge of dyslexia, Tailor and Coyne (2014:2) are of the view that ‘the awareness and knowledge held by teachers about dyslexia does affect their ability to help a learner in the classroom’. Another study was conducted by Elias (2014) in New Zealand that sought to examine the nature of teacher knowledge about dyslexic learners. The results further revealed the teachers’ lack of knowledge on what modality of teaching should be employed and resources that should be used.

As already indicated above, few studies have been conducted in the developing countries on the learning challenges faced by learners with dyslexia. One of the prominent studies is a study conducted by Shetty and Sanjeev Rai (2014) in India. The results of their study concluded that only 1 in 3 teachers had adequate knowledge of dyslexia. Alawadh (2016) conducted a similar study on teachers’ perceptions of the challenges related to provision of services for learners with specific learning difficulty (dyslexia) in Kuwait. The results of this study revealed that dyslexia was conceptualised differently by teachers in Kuwait as compared to their counterparts in the developed countries. The overall conclusion was that teachers were disempowered, lacked training and did not have sufficient knowledge of dyslexia or how to provide suitable early interventions. A number of studies conducted in the developing countries reveal that teachers have minimal readiness to identify learners with dyslexia (Abraham 2014: Peires et al. 2021).

In the South African context, the Department of Basic Education (2011:2) states that teachers must have a clear understanding of the needs of all learners, including those with special educational needs and be able to use and evaluate distinctive teaching approaches to engage and support them. However, as mentioned earlier, inadequate teacher training may leave teachers ill-equipped to meet this requirement. Literature identifies early interventions to strengthen the language foundations for reading as important. The key aspects related to early interventions are that they require trained practitioners (Hulme & Snowling 2016), that interventions should occur in the early years of primary schooling (Khaliq et al. 2017; Sako 2016; Torgesen, Foorman & Wagner 2007) and that interventions should include issues related to learning styles (Mortimore 2008).

Based on the literature cited above, the authors of this article are of the view that the developed countries seem to have made progress as far as addressing the learning challenges faced by learners with dyslexia. The development of policies and frameworks for understanding dyslexia bears testimony to this claim. However, in the developing countries, the literature reviewed does not demonstrate such efforts by governments.

## Method

### Research design

This research employed both the quantitative and qualitative research methods, which is also known as mixed methods research. Creswell (2014:40) states that ‘mixed methods employ strategies of inquiry that involve gathering data either simultaneously or sequentially to best understand research problems’. The mixed methods approach was deemed relevant for this study because it allowed the researchers to understand contradictions between quantitative and qualitative findings. One method complements the other. The qualitative method allowed the data to be collected deeply as it allows clarity seeking to be asked at the place with the participants. Quantitative data brings in a more balanced view to the study by present quantitative data. The primary reason for combining quantitative and qualitative approaches is that it allows for more comprehensive and synergistic use of data in offering a better understanding of research problems and complex phenomena than either approach could provide on its own (Fetters & Freshwater 2015:44).

Because the study relied on the teachers’ awareness and knowledge of dyslexia, the phenomenological design was deemed relevant. According to Cohen, Manion and Morrison (2007), phenomenology is a:

[*T*]heoretical point of view that advocates the study of direct experience taken at face value and one, which sees behaviour as, determined by the phenomena of experience, rather than by an external, objective and physically described reality. (p. 22)

Therefore, phenomenology allowed the participants to present their voices about their awareness and knowledge of dyslexia.

### Participants

The sample consisted of 30 government school primary teachers from two schools in Gauteng province, South Africa. These teachers were purposively selected, as language teaching was one of their subjects. In addition, these teachers were selected on the basis that they had learners who experienced reading difficulties in their classrooms, not necessarily in the year in which the study was conducted but even in the previous years. The teachers were teaching Grade 1 to Grade 5. The sample of teachers consisted of 25 woman and 5 men with their ages ranging from 23 to 63 years. The mother tongues of the respondents were Afrikaans, Setswana, isiZulu and Sepedi. These teachers were teaching their mother tongues as well as English First Additional Language (EFAL) and their qualifications ranged from a diploma to an honours degree.

### Instrumentation

A questionnaire was used to collect data in this study. Nduku (2020:295) defines a questionnaire as ‘a research device or instrument that is made up of a series of questions which are closed-ended or open-ended’. The questionnaire for this study consisted of 20 True or False statements, 10 closed-ended questions and 12 open-ended questions that were used to gather data from the respondents. With regard to closed-ended questions, a 5-point Likert-type scale was used in which respondents specified their level of agreement to a statement typically in the following five points: (1) Strongly disagree or SD, (2) Disagree or D, (3) Not sure or NS, (4) Agree or A, and (5) Strongly agree or SA. This was adopted to suit the purpose of this study, which addresses a sensitive topic, whereby Cohen, Marion and Morrison (2011) assert that a questionnaire has the ability to preserve anonymity and deal with sensitive areas of study. As already mentioned, the open-ended aspect of the questionnaire consisted of 10 open-ended questions. In this study, the researchers collected quantitative data by asking closed-ended questions and qualitative data by asking open-ended questions. The reason for combining open-ended questions with closed-ended questions is that the closed-ended questions have a limited set of possible answers like true or false. The open-ended questions allowed the participants to answer in any manner they chose. Moreover, the open-ended questions afforded respondents the ability to give longer answers and yielded more insights because respondents were able to elaborate their responses.

### Data analysis

After the quantitative data were collected from the sample participants, the researchers employed descriptive statistics to analyse, interpret the data, and give meaningful analysis and discussions. Descriptive statistical tools such as percentages, tables, graphs and figures were employed to strengthen the findings of the study through SPSS Version 22 and Excel data analyser 4. According to Maree (2007), qualitative data analysis involves working with data, organising them, categorising them into manageable units, synthesising them, searching for patterns, and discovering what is important and coming up to reliable conclusions. In this study, the researchers used content analysis to analyse the qualitative data solicited by open-ended questions. Columbia University (2019) defines content analysis as a research tool that is used to determine the presence of certain words, themes, or concepts within some given qualitative data. They further point out that sources of data for content analysis could be from interviews, open-ended questions, field notes, and so on. The researchers sifted through the open-ended responses one by one and decided what codes were the best fit. This was followed by coding the data into manageable code categories for analysis. In the context of this study, coding is the process of assigning codes to the open-ended answers. Individual responses were assigned a numerical code. Each code represented a segment consisting of similar responses.

### Ethical considerations

Ethical clearance to conduct this study was obtained from the Research Ethics Committee of the Tswane University of Technology, reference number: 2013.09/008.

## Results

### Quantitative data

Question one enquired about the teachers’ general level of awareness and understanding of the concept ‘dyslexia’. Below are the quantitative findings that seek to provide answers to this question.

Items in Table 1: (Items 1, 3, 13 and 19) show broad definitions of the concept ‘dyslexia’ and these responses answer Question 1. This is depicted by the word ‘is’ which acts to define a concept in response to the question: what is dyslexia or what causes dyslexia?

**TABLE 1:** Teachers’ broad understanding of the concept ‘dyslexia’.

Statement	Incorrect answers	% of incorrect answers	% of correct answers
1. Dyslexia is a neurological disorder.	07	23	77
2. Dyslexic readers demonstrate weak phonological processing skills.	09	30	70
3. Dyslexia is limited to the English-speaking population.	04	13	87
4. If you just give learners enough time, they will outgrow dyslexia.	03	10	90
5. A person with dyslexia will never learn to read.	03	10	90
6. Learners with dyslexia have problems in learning letters of alphabet.	01	3	97
7. Learners with dyslexia experience repeated erratic spelling errors.	01	3	97
8. In some learners, dyslexia can affect writing, maths and language.	0	0	0
9. Learners with dyslexia have trouble recognising letters and matching letters to sounds.	01	3	97
10. Learners with dyslexia avoid reading, both aloud and to themselves.	03	10	90
11. Learners with dyslexia do not read at the expected level.	03	10	90
12. Learners with dyslexia have a below average intelligence	0	0	0
13. Dyslexia is hereditary.	09	30	70
14. Physicians can prescribe medication to help dyslexia.	02	7	93
15. Multisensory instruction can be useful for learners with dyslexia.	03	10	90
16. Learners with dyslexia struggle with learning new words.	03	10	90
17. Learners with dyslexia have trouble recognising whether two words rhyme.	05	17	83
18. In school, dyslexia affects the learners’ performance in reading (not in maths, social studies, etc.).	07	23	77
19. Dyslexia is a lifelong condition that makes it difficult for people to read.	04	13	87
20. Every struggling reader is dyslexic.	01	3	97

This question sought to gauge how much the participants knew about dyslexia on a general level. Their responses were anticipated to form a build-up to questions 2, 3 and 4. The assumption was that if the participants had some basic knowledge about dyslexia, they would be able to apply such knowledge when dealing with learners with dyslexia. This question was also anticipated to check how much misinformation or myths the participants had about dyslexia as this would inform their prejudice against learners with dyslexia. Table 1 shows these responses. For example, Table 1 shows that 77% of the participants knew that dyslexia was a neurological disorder. Eighty-seven per cent of the participants dismissed the myth that dyslexia was limited to the English-speaking population. However, the researchers were concerned about 13% of the participants who accepted this myth as reality. Table 1 further reveals that 70% of the participants were aware that dyslexia was hereditary. This information is important for the participants because it might facilitate collaboration between the school and the learner’s home to collectively find a strategy that might assist the learners with dyslexia. Table 1 also revealed that 87% of the participants knew that dyslexia was a lifelong condition that made it difficult for people to read.

This awareness was important so that the participants did not give up hope on the learners with dyslexia when they did not master reading as other learners did. This was also likely to assist participants so as to not set unobtainable goals for themselves. For ease of reading Table 1, items 2, 5–12, 16–18, and 20 show the participants’ understanding of how dyslexia affects teaching and learning. The researchers observed that 30% of the participants were not aware that dyslexic readers demonstrated weak phonological processing skills. Table 1, items 4, 14 and 15 further shows the participants’ understanding of solutions that could be undertaken to support learners with dyslexia. In summary, the findings showed that most participants were clear about the reading difficulties that learners with dyslexia had, in terms of language learning. The responses were as follows: Readers with dyslexia demonstrated weak phonological processing skills (70%), struggled to read (90%); had problems in learning letters of alphabet (97%); experience repeated erratic spelling errors (97%); had trouble recognising letters and matching letters to sounds (97%); avoided reading, both aloud and to themselves (90%) and that they did not read at the expected level (97%). To show the participants’ understanding of dyslexia 90% of them disagreed with the false statements that ‘giving learners enough time would allow them to outgrow dyslexia’. Ninety-three per cent of the teachers also did not agree ‘medication can help learners with dyslexia’. Ninety per cent agreed that multi-sensory instruction could assist learners with dyslexia during the process of learning. All these statements prove that most participants had a clear awareness and understanding of dyslexia.

**FIGURE 1:**
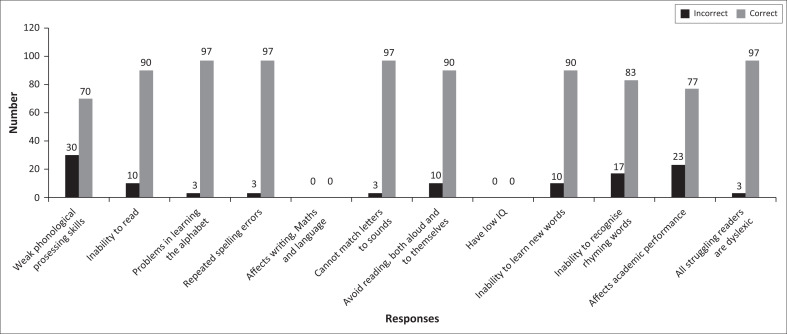
Teachers’ understanding of the limitations caused by dyslexia to learners.

**FIGURE 2:**
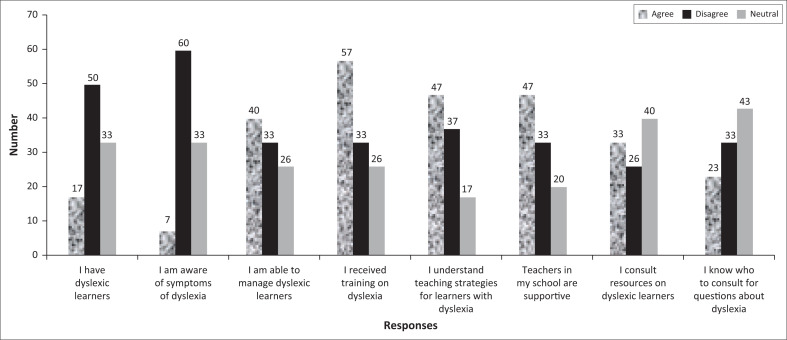
The way teachers navigate teaching and learning within reading difficulties brought about by dyslexia.

Question two of the study focused on the participants’ understanding of limitations brought about by dyslexia in teaching and learning and the possible solutions thereof. Figure 1 reveals that the majority of participants demonstrated an understanding of solutions on how to support learners with dyslexia in the classroom. Moreover, the participants’ responses depicted their understanding of reading difficulties that learners with dyslexia had and perhaps their own limited skills as teachers, in teaching such learners. This, in turn, showed how the participants were able or unable to cope with teaching learners with such reading difficulties and available solutions. In addition, responses to this question show teachers’ understanding of how difficult or easy it is to resolve reading difficulties brought about by dyslexia. Figure 2 shows the participants’ responses regarding such solutions.

### The way teachers navigate teaching and learning within reading difficulties brought about by dyslexia

Question three of the study focused on the way teachers navigated teaching and learning within reading difficulties brought by dyslexia. This question sought to understand the participants’ daily practices and support when dealing with dyslexia in their classrooms. This was depicted by the letter ‘I’ and ‘my’. The analysis is shown in Table 2.

**TABLE 2:** Interest in further training on dyslexia.

Question	Agree		Disagree	Neutral
	*n*	%		
I am interested in additional dyslexia training.	30	100	0	0

The findings showed that only 17% of the participants had learners with dyslexia in their classes, 50% currently did not have such learners in their classes while 10% did not respond to the question. This, however, refers to the year of the research but does not mean that these participants had never had or never would have learners with dyslexia in their classrooms. The findings further show that 7% of the participants were aware about the symptoms of dyslexia, while 93% said that they were not aware and/or not sure.

The majority of participants (57%) indicated that they had received training on the topic of dyslexia; 40% could manage learners with dyslexia in their classes; 47% understood the possible teaching strategies to accommodate learners with dyslexia (47%); 47% said that teachers in their schools came together and shared ideas, strategies, and materials to support learners with dyslexia. For further support, a low 33% said they consulted resources regarding readers with dyslexia while the majority (40%) were neutral. Only 23% said they knew whom to consult when they had questions about dyslexia and learners with dyslexia; 33% responded that they did not know whom to consult while 43% were neutral. Figure 2 shows that the majority of participants were aware of the symptoms of dyslexia and had received training on dyslexia.

### The teachers’ interest in being skilled to cope with dyslexia in their classrooms

This fourth question sought to gauge the level of interest that participants had in acquiring further skills in dealing with dyslexia in their classes.

Table 2 reveals that all the participants indicated that they were willing to have additional dyslexic training. The responses to this question showed how the participants were proactive in addressing their shortfalls in a class with learners with dyslexia. This question may even point towards a vital gap that the Department of Basic Education needs to address in Alternative Education.

### Qualitative data

The first question focused on the level of teachers’ general awareness of the concept ‘dyslexia’. As already mentioned above, 30 participants responded to the closed-ended questions as well as the open-ended questions. Generally, the participants showed that they were aware and had some knowledge about dyslexia. Some participants mentioned that although they did not have such learners in their classrooms at the time the study was conducted, in the previous years they used to have learners with dyslexia and therefore had experience about them. To substantiate this, Teacher 4 said the following:

‘A learner with dyslexia is a learner who struggles with reading. They cannot remember the words; they will spell it every time. This is a learning disfunction. There is no medicine for this. They cannot spell, they struggle with spaces between words. Everything is in one sentence from the top of the page till the last word on the page.’ (Grade 1 teacher, female, 42 years)

Responding to the same question, Teacher 1 said:

‘For learners with dyslexia, letters and words look like they are moving/jumping/turning. The learner struggles to make sense of it. It takes a lot of effort and concentration to make sense of the word. Because of the letters moving, in their brain, it gives a lot more possibilities of what the word can look like. Then the learner has to find/identify the correct word. After this process, the learner can only then read/pronounce it or write it.’ (Grade 3 teacher, female, 38 years)

On the same question, Teacher 3 responded this way:

‘It is a disorder that affects a person’s ability to write and read. This is because of constant letter movement. For example, the reader finds that words “flow”; “move” or blur on the paper. Sometimes when writing they will experience letter confusion (n/u; b/d). They often feel shy about their situation although there is nothing wrong with their abilities.*’* (Grade 2 teacher, female, 44 years)

The second question focused on the teachers’ understanding of reading difficulties brought about by dyslexia in teaching and learning. In this regard, the participants showed some experience in teaching learners with dyslexia in the classrooms. Some participants emphasised that as far as intelligence is concerned, these learners are not different from other learners but struggle to read and write.

Responding to this question, Teacher 1 mentioned:

‘Their work speed is very slow. They can come across as disorganised. Sometimes are quiet learners. They do not want to read aloud or give answers in front of other learners. Some of these learners can have behavioural problems due to frustration or feeling “dumb/stupid”. They are also not able to follow the instructions.*’* (Grade 3 teacher, female, 38 years)

Teacher 6 mentioned the following:

‘The learners with dyslexia have a challenge with the writing of letters or numbers e.g. d instead of b. They can have below average to above average intelligence. They do not look different from other learners.’ (Grade 5 teacher, male, 32 years)

Responding to the same question, Teacher 5 said:

‘These learners feel frustrated and unsure of themselves. They view themselves as people who fail to perform to expectations especially on written platform but could excel on verbal platform. These children miss out on things that their peers enjoy in life.’ (Grade 4 teacher, female, 54 years)

The third question focused on how the participants navigate teaching and learning within the reading difficulties brought about by dyslexia. Some participants’ responses indicated that they were doing something to assist the learners with dyslexia. Some respondents indicated that they used visual materials while others pointed out that they used clay to develop the letters of the alphabet together with the learners with dyslexia. These participants believed that learners with dyslexia performed better in hands-on activities. They believed that learners with dyslexia were kinaesthetic and therefore, using a teaching style that matches their learning style would facilitate their learning potential.

Responding to this question, Teacher 4 said:

‘I do my best to be as concrete and visual as possible. I use verbal cues or pictures that can help the learner with dyslexia understand. Sometimes, I build words with clay and also write in the sand. What I have observed through experience is that these learners learn better when they are doing something with their own hands. Educators and parents need to educate themselves about how to provide support to dyslexic learners as some form of intervention.’ (Grade 1 teacher, female, 42 years)

Teacher 2 provided a similar response when she mentioned that:

‘We have to help the learners with dyslexia by giving them the necessary support. All learning material on CDs and Reader and Scribe for all formal assessments. These learners should be allowed to take reading material home so that their parents can also play a role in teaching them reading.’ (Grade 4 teacher, male, 31 years)

The final research question sought to know if the participants were interested in dyslexia training. All participants indicated their willingness to receive training to support learners with dyslexia. Many of them indicated that they never had pre-service training on dyslexia but had some in-service training. Therefore, they concluded that their training on dyslexia was limited. One of their reasons to need more training on dyslexia was that they wanted to be able to distinguish a learner with dyslexia from other learners who have normal reading challenges. The participants also indicated that it was difficult for them to identify the learners with dyslexia and they would appreciate any support in this regard. To this end, Teacher 2 said:

‘I am willing to receive training on learners with dyslexia to broaden my knowledge and be able to assist the learners with dyslexia. I want to help them to reach their optimal potential.’ (Grade 4 teacher, male, 31 years)

Responding to the same question, Teacher 7 said the following:

‘I would like to learn how to identify these learners. Maybe alternative test methods can assist us because when they write tests, they do not finish within the duration of the test given to all the learners. They always need more time. In our school, we have many learners who are dyslexic. Therefore, any opportunity, such as training would assist us.’ (Grade 5 teacher, male, 47 years)

Also responding to the same question, Teacher 3 pointed out:

‘Yes, I need training to be able to distinguish between whether the reading challenge is dyslexia or another reading challenge. Secondly, I want to be better equipped to help the learner achieve his/her potential.*’* (Grade 2 teacher, female, 44 years)

## Findings and discussion

The study sought to evaluate the awareness and knowledge of dyslexia among public primary school teachers in Gauteng. The study was triggered by the government’s adoption of the inclusive education policy in order to address the barriers to learning in the education system. This move has been viewed as not practical in the South African classrooms because some scholars, such as Prinsloo (2008) and Peters (2007) argue that learners with disability such as learners with dyslexia remain vulnerable.

As indicated earlier, this study is linked to the implementation of the inclusive education policy. We highlighted the importance of the role of teachers’ empowerment or development in order to realise the dream of inclusive education as well as effective management of learners with disability. Specifically, the first question focused on the level of teachers’ general awareness of the concept ‘dyslexia’. Generally, the teachers showed that they were aware and had some knowledge about dyslexia. Some teachers mentioned that although they had not received any pre-service training, they managed to teach learners with special education needs. Although it was highlighted in the literature review section that teachers’ awareness and knowledge of dyslexia is significant for teachers, several studies have examined teacher awareness and knowledge of dyslexia and generally found weaknesses in some areas of awareness and knowledge and strengths in others (Joshi, Washburn & Kahn-Horwitz 2016; Elias 2014; Furnham 2013; Knight 2018). Another study that contradicted the findings of the current study about teachers’ awareness and knowledge of dyslexia is the one conducted by Khaliq et al. (2017) in Pakistan. Their findings revealed that teachers of the elementary schools from Lahore were not aware of the term dyslexia and only few of them were able to identify and manage it in their classrooms. These studies contradict the findings of the current study. This can be attributed, perhaps, to the different contexts and methodologies implemented.

The second research question focused on the participants’ understanding of reading difficulties brought about by dyslexia in teaching and learning. In this regard, the participants showed some experience in teaching learners with dyslexia in the classrooms. Some participants emphasised the fact that as far as intelligence is concerned, the learners with dyslexia were not different from other learners but struggled to read and write. The majority of participants demonstrated an understanding of solutions on how to teach learners with dyslexia in the classroom. Moreover, the participants’ responses depicted their understanding of reading difficulties that learners with dyslexia had and perhaps their own limited skills as teachers, in teaching such learners. The fact that teachers had limited skills to cope with learners with disabilities in an inclusive classroom has been observed by various authors. For example, Dalton, Mackenzie and Kahonde (2012) discovered that in South Africa, the implementation of inclusive education is limited by a lack of teachers’ skills and experience in customising the curriculum to suit a wide range of learning needs. This could be one of the reasons why all teachers in the current study yearned for training on how to cope with learners with special education needs.

The third question focused on how the participants navigated teaching and learning within reading difficulties brought about by dyslexia. Some participants’ responses indicated that they were doing something to assist the learners with dyslexia. Some participants indicated that they used visual materials while others mentioned that they used clay to develop the letters of alphabet together with the learners with dyslexia. These participants observed that learners with dyslexia performed better in hands-on activities. They believed that learners with dyslexia were kinaesthetic and therefore, using a teaching style that matches their learning style would facilitate their learning potential. This finding supports Adewumi et al. (2019) who concluded that teachers claimed to assist the learners with special education needs despite the fact that they did not receive any pre-service training. In this situation, it could be that the in-service workshops conducted yielded positive results. Chiappetta-Swanson and Watt (2011:4) also mentioned instances in which teachers manage the situation without any proper training when they refer to supervisors who are not trained as ‘muddling through’. Similarly, in this case teachers manage to cope more or less satisfactorily despite the lack of expertise and/or equipment.

The final research question sought to know if participants were interested in dyslexia training. All the participants indicated their willingness to receive training in dyslexia. Many of them indicated that they never had pre-service training on dyslexia but had some in-service training. Therefore, they concluded that their training on dyslexia was limited. This finding is in line with Knight’s (2018) finding that declared that evidence-based teacher training, which informs teachers of the up-to-date research on the biological, cognitive, and behavioural aspects of dyslexia, is essential to combat misconceptions and ensure that teachers have more nuanced and informed understandings of dyslexia. Abd Rauf et al. (2018) have also highlighted the importance of teacher training on dyslexia. They alluded to the fact that learners with dyslexia needed trained teachers as well as a supportive school community teacher training is important for the early diagnosis of learners with dyslexia. Also, while highlighting the importance of teacher training to understand dyslexia, Hulme and Snowling (2016) argued that educational interventions for reading and related learning disorders are effective when delivered by trained practitioners.

## Conclusion

This article has raised several matters related to evaluating the awareness and knowledge of dyslexia among public primary school teachers. To this end, it was mentioned in the introduction that learners with dyslexia needed trained teachers as well as a supportive school community. Regarding the teachers’ awareness and knowledge of dyslexia, it is evident that the teachers participated in this study were aware and had some knowledge about dyslexia although they did not receive any information about dyslexia in their pre-service training. However, this finding did not imply that the teachers were totally oblivious to the classroom practice that may be helpful to dyslexic learners. As such, we concluded that there is room for improvement as far as learning how to teach learners with dyslexia in the classroom is concerned. The findings of the study further revealed that most teachers demonstrated some understanding of the solutions on how to deal with learners with dyslexia in the classroom although their coping strategies with these learners were, in many instances, limited. The findings also revealed that the teachers navigated teaching and learning within reading difficulties brought about by dyslexia. To this end, some teachers indicated that, informed by their experience, the learners with dyslexia found learning, especially, reading better, when they used clay to develop the letters of alphabet together with the dyslexic learners. Thus, some teachers observed that the learners with dyslexia preferred a kinaesthetic learning style. It means that they learn by doing something with their hands. Finally, the study revealed that all the teachers were interested in dyslexia training. The willingness of the teachers to receive training could be motivated by their passion to assist the leaners with dyslexia and their understanding that they need more specialised training in order to be specialised teachers necessary for inclusive education.

## Recommendations

In the context of the data collected and the findings made, the researchers recommend that, in order to enhance teachers’ awareness and understanding of dyslexia in the public primary schools, teacher training institutions in South Africa should provide adequate and relevant pre- and in-service training courses on the pedagogy of teaching learners with dyslexia. We felt that dyslexia should form part of the reading component of the teacher training tertiary institutions. No participants in the study mentioned the relationship between the teachers and the parents of learners with dyslexia. We felt that a strong bond between the two parties should exist so that teachers report the progress and challenges faced by learners with dyslexia to their parents. In return, the parents should play their role by assisting and motivating these learners to read at home. The literature review section pointed that early identification of dyslexia could improve the learning opportunities for the learners with dyslexia. Once the teachers and the parents identify the challenge at an early stage, other stakeholders like speech therapists and psychologists should also be involved at an early stage.

The participants in the study complained about their lack of skills on how to identify and assess the learners with dyslexia. This is a cause for concern because these learners do not take the time taken by the ‘normal learners’ when it comes to classroom activities and examinations. Therefore, the teachers should be made aware that these learners need extra time in order to finish their tasks. We also recommend that teachers give the remedial support to assist learners according to their identified reading difficulties. This move is likely to allow the learners with dyslexia to catch up with their peers. Finally, we recommend a collaborative effort among all stakeholders and adequate training of teachers to ensure effective support for learners with dyslexia.
