# Corrigendum: Evaluating the awareness and knowledge of dyslexia among primary school teachers in Tshwane District, South Africa

**DOI:** 10.4102/ajod.v12i0.1079

**Published:** 2023-05-09

**Authors:** Mary M. Makgato, Monicca Leseyane-Kgari, Madoda Cekiso, Itani P. Mandende, Rose Masha

**Affiliations:** 1 Department of Applied Languages, Faculty of Humanities, Tshwane University of Technology, Pretoria, South Africa

In the published article, Makgato, M.M., Leseyane-Kgari, M., Cekiso, M., Mandende, I.P. & Masha, R., 2022, ‘Evaluating the awareness and knowledge of dyslexia among primary school teachers in Tshwane District, South Africa’, *African Journal of Disability* 11(0), a807. https://doi.org/10.4102/ajod.v11i0.807, the name of one of the authors was incorrectly spelled in the reference for Taylor and Coyne (2014) as Tailor. It should be Taylor.

The authors apologise for this error. The correction does not change the study’s findings of significance or overall interpretation of the study’s results or the scientific conclusions of the article in any way.

